# CheS-Mapper - Chemical Space Mapping and Visualization in 3D

**DOI:** 10.1186/1758-2946-4-7

**Published:** 2012-03-17

**Authors:** Martin Gütlein, Andreas Karwath, Stefan Kramer

**Affiliations:** 1 Department of Computer Science, Albert-Ludwigs-Universität Freiburg, Freiburg im Breisgau, Germany; 2 Institute for Computer Science, Johannes Gutenberg-Universität Mainz, Mainz, Germany

## Abstract

Analyzing chemical datasets is a challenging task for scientific researchers in the field of chemoinformatics. It is important, yet difficult to understand the relationship between the structure of chemical compounds, their physico-chemical properties, and biological or toxic effects. To that respect, visualization tools can help to better comprehend the underlying correlations. Our recently developed 3D molecular viewer CheS-Mapper (Chemical Space Mapper) divides large datasets into clusters of similar compounds and consequently arranges them in 3D space, such that their spatial proximity reflects their similarity. The user can indirectly determine similarity, by selecting which features to employ in the process. The tool can use and calculate different kind of features, like structural fragments as well as quantitative chemical descriptors. These features can be highlighted within CheS-Mapper, which aids the chemist to better understand patterns and regularities and relate the observations to established scientific knowledge. As a final function, the tool can also be used to select and export specific subsets of a given dataset for further analysis.

## Background

The area of quantitative structure-activity relationship (QSAR) modeling, an active field in chemoinformatics, includes numerous approaches, many based on numerical features, structural fingerprints, or sub-graph mining [1-3]. Commonly, the employed features are then used with machine learning approaches like support-vector-machines or decision trees to predict activities for previously unseen compounds. However, many algorithms do not convey information about the relevance of features with respect to particular activity values in the learnt models. Visualization can be vital to examine the dataset for possible interdependencies between compound structures, compound features, and endpoint values. In the work described here, we present a general, interactive, and open-source application called CheS-Mapper, for the inspection of chemical datasets of small molecules, enabling scientific researchers to experiment with the compounds and their features. Our application visualizes compound structures and features in a virtual 3D space. The tool detects subgroups (clusters) within the data, and can be employed to analyze the data to find possible structure-activity relationship (SAR) information.

We give a short overview of existing visualization methods. The probably most commonly used tool to explore a small molecule dataset is the chemical spreadsheet. It lists all compounds, usually with a small picture showing its 2D structure, and other properties in one single table. There are several commercially available spreadsheet programs [4-6]. The open-source workframe Bioclipse [7] has also a built in spreadsheet tool. These tools often incorporate plug-ins to show the 3D structure of single compounds, and plotting functions like heat-maps for compound properties. A common plotting method, that is frequently available within chemical spreadsheet programs as well, is based on multi-dimensional scaling [8]. This technique reduces the high-dimensional input feature space in order to create a 2D dot plot, where each dot corresponds to a compound. The Structure-Activity Landscape Index (SALI) [9] can detect compound pairs that are structurally very similar, but differ largely in their activity value. The freely available program creates graphs, where each node corresponds to a compound and each edge correspond to a compound pair with high SALI value. A related approach has been taken by a method called Similarity-Potency Trees [10], by visualizing SAR information present in a dataset as trees. The approach derives a 2D tree, that connects two compound-nodes solely if they are structurally similar (based on fingerprints), not using the activity value. Instead the activity values are used to color the nodes accordingly, which helps to identify structural differences that cause discontinuous changes in activity levels. The SPT program is also freely available. It is, however, limited in the sense that the user has to provide the exact features, e.g. pre-calculated fingerprints.

Compared to existing methods, the presented CheS-Mapper application is a unique combination of clustering, multidimensional scaling, and 3D viewer. The user works directly with the 3D structure of each compound, instead of substituting compounds with dots or nodes. Within CheS-Mapper, compounds are mapped into 3D space according to their similarity. It is a generic tool, as it is up to the user to define similarity by choosing the features that are employed within the mapping process. CheS-Mapper requires no installation, it accepts a wide range of chemical datasets, it has a wizard guiding through the mapping process, and it has an intuitive 3D viewer for exploring chemical space. Well-established chemical and data-mining libraries are integrated to offer many algorithms for expert users.

## Methods

**Figure 1:**
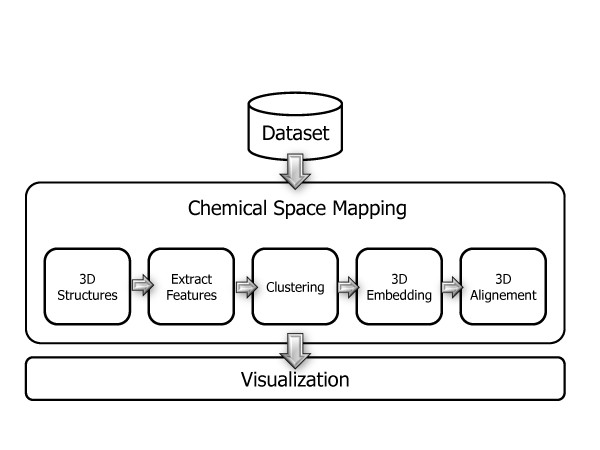
**The CheS-Mapper Workflow**. The CheS-Mapper application workflow is divided into two main parts: Chemical Space Mapping (can be configured with a wizard) and 3D Visualization.

The CheS-Mapper program is a graphical application that can be used to visualize chemical datasets of small molecules (compounds). The application is divided into two main parts, namely *Chemical Space Mapping *and *Visualization*. The overall workflow can be seen in Figure 1.

In the first part, the Chemical Space Mapping, the molecules in the dataset are preprocessed. The compounds are grouped together into clusters and embedded into 3D space. The user has to select the features of the compound that are used as input for the clustering and the embedding algorithms. The features employed for this can either be features already precomputed in the original dataset or can be computed by the application. A range of chemical descriptors or structural features is available, as well as various clustering and embedding algorithms. Finally, the compounds of each cluster can be aligned according to common substructures. The preprocessing is described in more detail in the following section.

The second part of the application, the visualization, is then used to explore the dataset in a virtual three dimensional space. Hence, the application is based on a molecular 3D viewer, that provides basic yet intuitive capacities like rotating and zooming. Special highlighting functions allow properties of the dataset to be visually highlighted. This includes highlighting of cluster assignments, compound features, endpoint values, and structural fragments. Furthermore, the compounds of each cluster can be superimposed to provide a better overview of the whole dataset, and to point out structural (dis-)similarities. Compounds and clusters of compounds can be deleted and exported in standard file formats for further analysis. The visualization is described in more detail in section "CheS-Mapper Viewer" below.

### Configuring Chemical Space Mapping with the CheS-Mapper Wizard

As the Mapping Process offers a wide variety of options, like which clustering algorithm to use, or which 3D embedding technique to employ, we have designed a dedicated wizard to ease the use of the application. Each step is well-documented and provides reasonable default settings to support the novice user. Expert users will appreciate that most algorithms are highly configurable. Overall, there are six steps in the wizard, each step is described in one of the following sections.

#### Load Dataset

**Figure 2:**
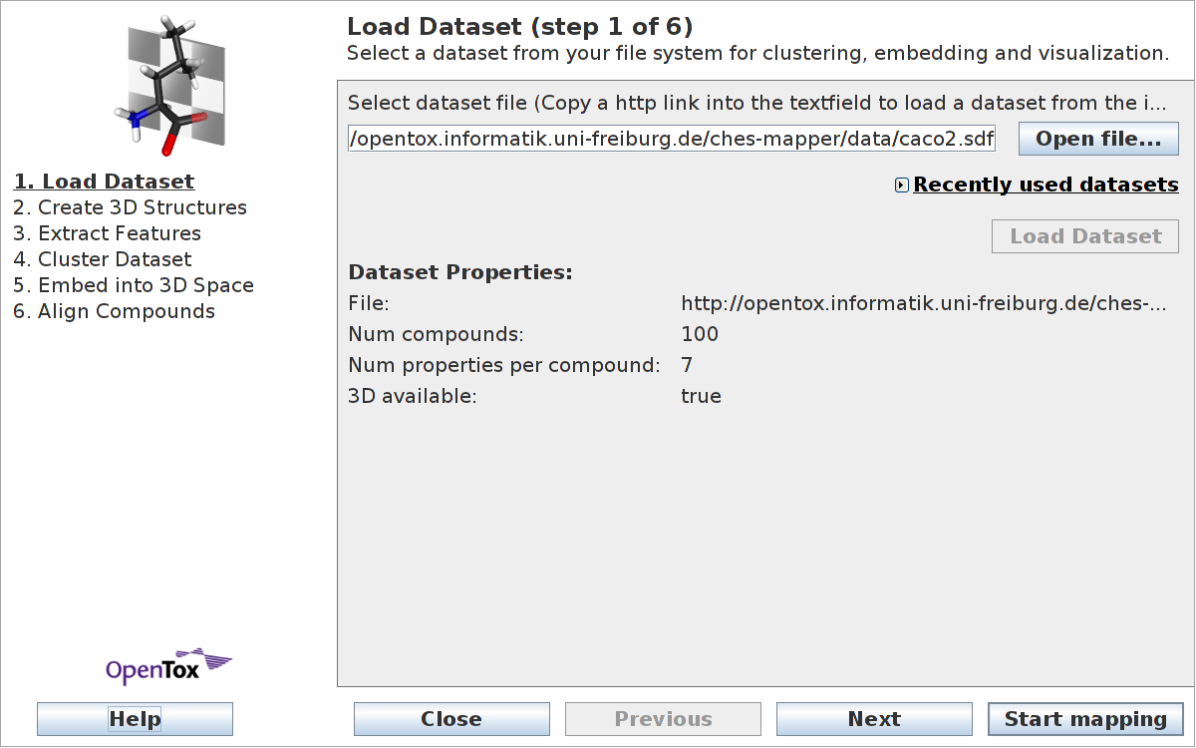
**Wizard Step 1: Load dataset**. A wide range of chemical file formats is supported. Users can load datasets from the local file system, as well as directly from the internet.

The dataset is selected in the first wizard step (see Figure 2). CheS-Mapper uses the CDK library (The Chemical Development Kit [11]) to read the dataset, which assures that a wide range of chemical formats is supported. (See additional file 1 for details on supported dataset formats and size.) When the dataset is entirely loaded, the wizard displays the number of compounds, the number of features for each compound, and a flag that indicates whether 3D structure information is already available in the dataset or has yet to be calculated (see the next section).

The wizard is able to load datasets from the local file system, as well as from the Internet. Additionally, datasets allocated at an OpenTox [12,13] dataset service can be used. The OpenTox project provides a API definition for a predictive toxicology framework, a publicly available dataset service is for example the AMBIT web service [14].

#### Create 3D Structures

**Figure 3:**
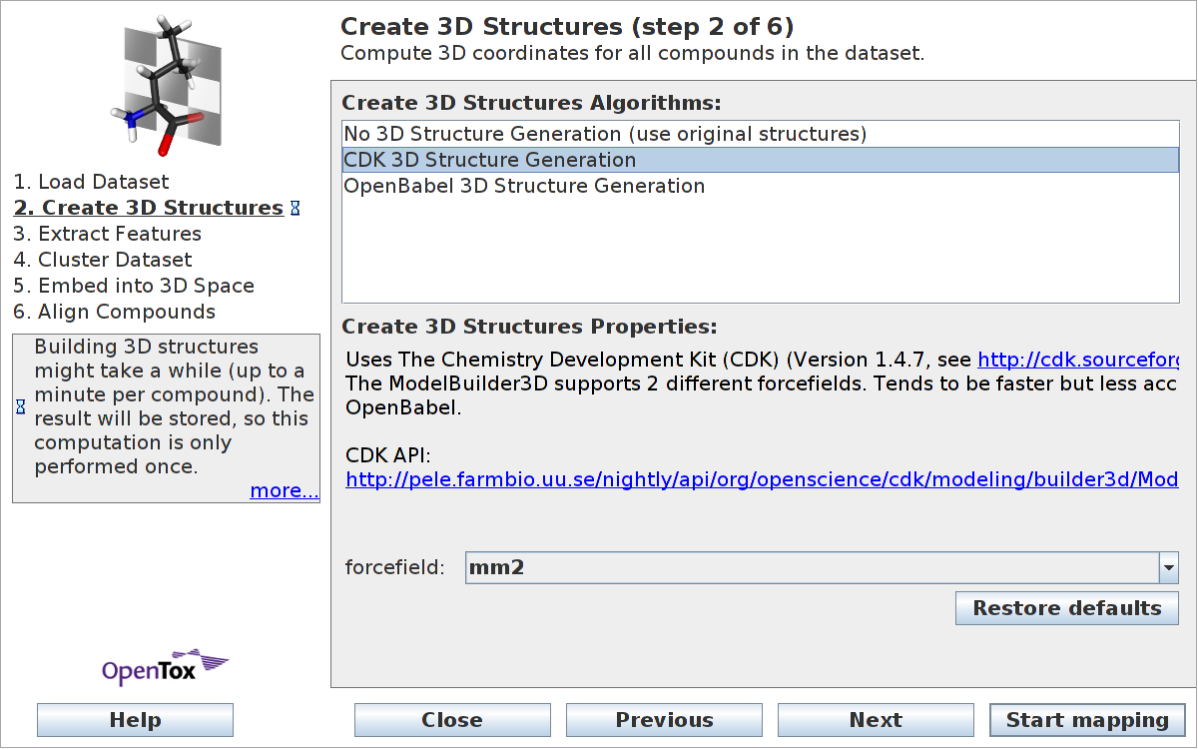
**Wizard Step 2: Create 3D Structures**. In cases, where the 3D structures of compounds is not already available, users can calculate these 3D structures with the chemical libraries CDK or Open Babel.

In this wizard step, three-dimensional (3D) structure can be calculated for the compounds in case it is not already present in the original dataset. The wizard window for the structure generation can be seen in Figure 3, where the 3D builders of the chemical libraries CDK and Open Babel [15] are exposed. The CDK structure builder allows to choose from two different force fields, MM2 [16] and MMFF94 [17] (Open Babel uses the latter). Building 3D structures is a time consuming process, and can sometimes take up to a few minutes per compound (see additional file 1 for runtime experiments). However, this has to be done only once for each dataset, the results are cached by the application and the structures are available for subsequent runs of the program. In case the 3D structure information is already provided in the original dataset, one can select *No 3D structure generation *(in case only 2D structure information is present and no 3D structures are calculated, 2D flat structures are later shown in the viewer).

#### Extract Features

**Figure 4:**
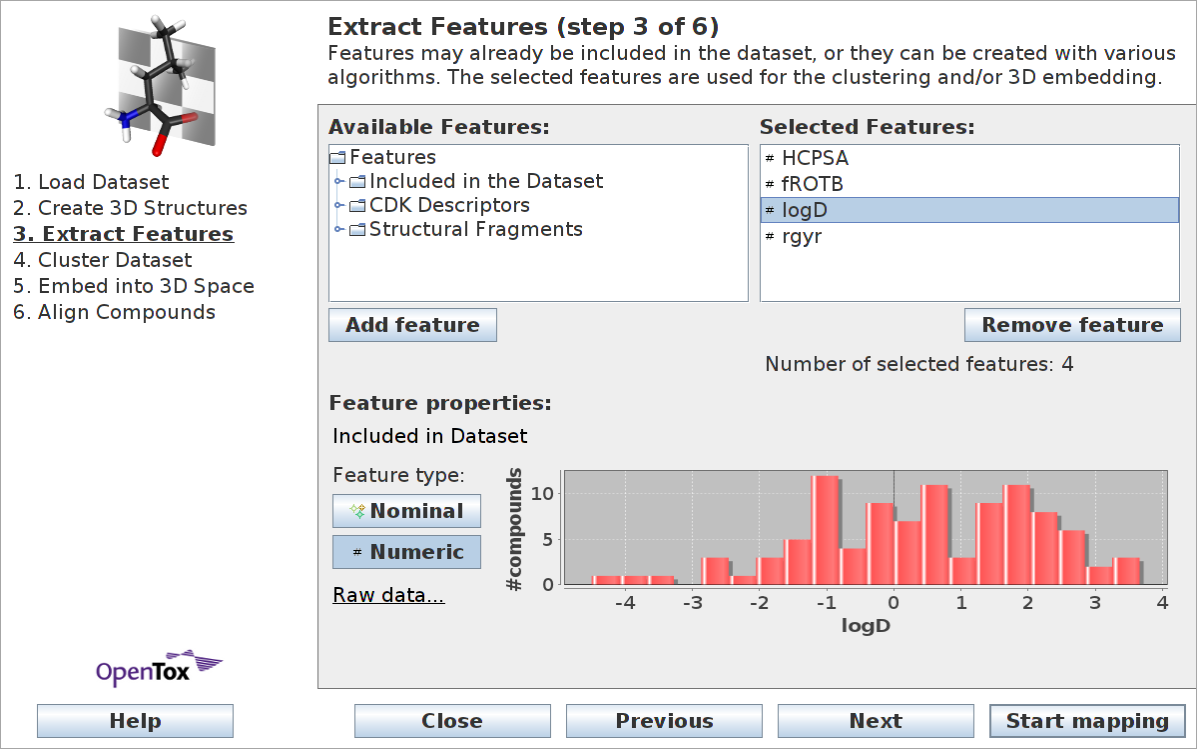
**Wizard Step 3: Extract features**. Users can select features that are precomputed in the dataset or can be computed by CheS-Mapper. Compounds with similar feature values are likely to be clustered together and are embedded closer to each other in 3D space.

In step three of the wizard, the user can select which features to employ in the subsequent steps (see Figure 4). These steps (clustering and embedding) are based on the selected features: compounds with similar feature values are likely to be clustered together into the same cluster. Likewise, these similar compounds are embedded closer to each other in 3D space, while compounds that have mainly different feature values will have 3D positions far from each other.

The CheS-Mapper application distinguishes between numerical and nominal features. Nominal features separate compounds into distinct categories (e.g. a nominal feature representing an activity could have values *active*, *moderately-active*, *inactive*). Numeric features have continuous floating point numbers as values (e.g. LogP or molecular weight). The software tries to guess the correct feature type when reading in the features present in the dataset. The feature type can be changed manually, if both feature types are possible (for example, when feature values are *0 *and *1*).

Within this wizard step, it is also possible to pre-compute and draw the feature values (see chart in Figure 4) in order to aid in the decision which features to select. Three different types of features are available:

• **Included in Dataset **Precomputed or experimentally measured properties that are available in the original dataset. Most QSAR datasets have a biological or toxic endpoint that is stored in the dataset. Often, the datasets also contain features that have been computed by some external software.

• **CDK Descriptors **The Chemical Development Kit provides a range of descriptor calculators that produce numerical features. This includes relatively simple features like molecular weight, or the number of rotatable bonds as well as sophisticated chemical descriptors like LogP or the van der Waals volume.

• **Structural Fragments **Structural fragments are encoded as SMARTS strings. The presence or absence of a particular structural fragment may play an important role, as the mode of action of a compound often is dependent on its structure. These fragments are called structural alerts. In the CheS-Mapper application, a structural fragment is encoded as a binary nominal feature with value *0 *or *1*: the feature has value *1 *if the compound contains the fragment (i.e. if the compound matches the SMARTS string), the value is *0 *if the fragment is not contained in the compound.

The available structural fragments in CheS-Mapper are either provided via SMARTS files, or can be mined with chemical libraries. The SMARTS files that are integrated into CheS-Mapper present results from recent publications (e.g., alerts that model drug induced phospholipidosis [18]). A user-defined SMARTS file can be added by clicking the *Add SMARTS file *button.

Furthermore, CheS-Mapper can directly derive structural fragments from Open Babel fingerprints. The four Open Babel fingerprint types are called *FP2*, *FP3*, *FP4*, and *MACCS. FP2 *is a enumeration of all linear fragments of size less or equal to *7 *atoms, the other three fingerprints use a predefined set of structural alerts.

The structural fragment mining can be configured by the user with various parameters: the minimum frequency defines a threshold for the number of compounds that must be matched by the fragment. A flag decides whether fragments should be skipped that occurring each compound. CDK or Open Babel can be selected as SMARTS matching software, whereas Open Babel is much faster and should be preferred. The Open Babel fingerprint based structural fragments can be mined with Open Babel only.

#### Cluster Dataset

The cluster settings are configured in wizard step 4. Clustering divides the dataset into subgroups. In general, compounds having similar feature values are grouped together in one cluster. Only features, that have been selected in the previous step are used as input to the clustering algorithm. Clustering provides the following benefits for the visualization: the user is given indication if there are natural subgroups within the dataset. The dataset is therefore easier accessible, as large datasets can hardly be shown on the screen at once. Furthermore, computing the maximum common subgraph inside a cluster can give further insights towards the structural similarity of the compounds.

**Figure 5:**
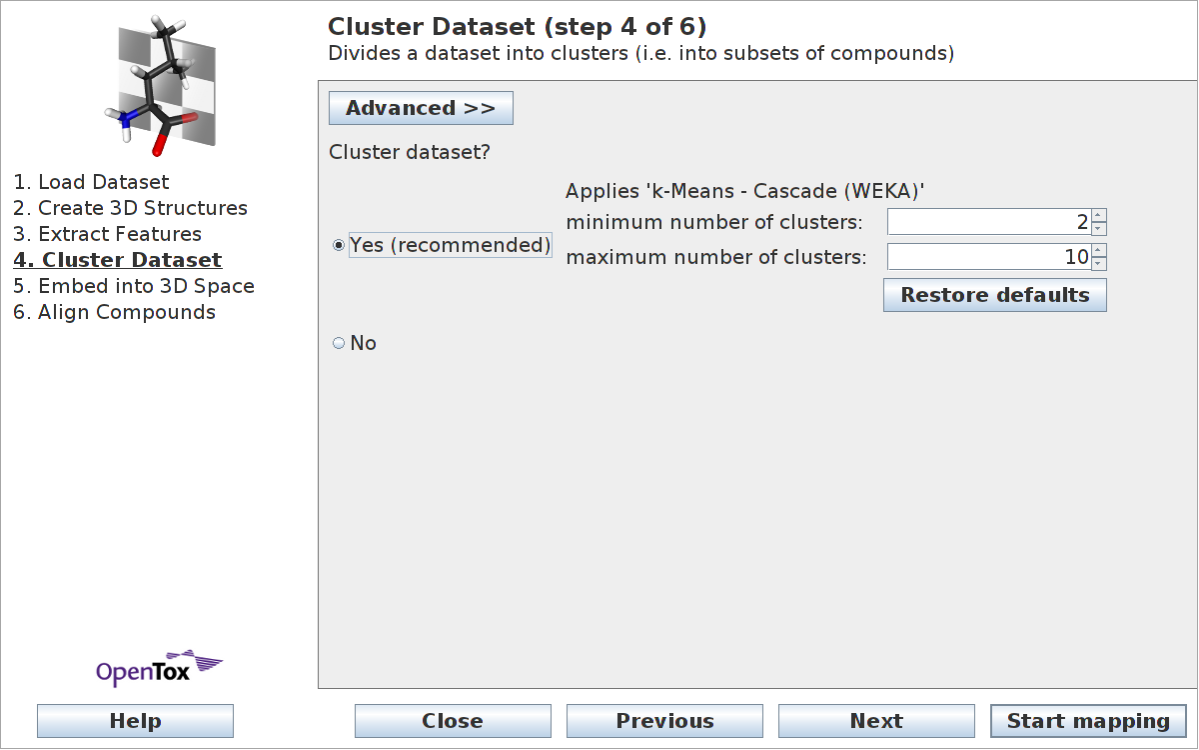
**Wizard Step 4: Cluster Dataset - simple view**. With the simple view, users can set the lower and upper bound on the number of clusters.

Figure 5 shows the *simple *view of wizard step 4. The user can set lower and upper bounds on the possible number of clusters (The Cascade *k*-Means algorithm is used for clustering, as described below). Alternatively, there is the choice to *not *to cluster the dataset. This is the only viable option if no features have been selected in the previous step.

**Figure 6:**
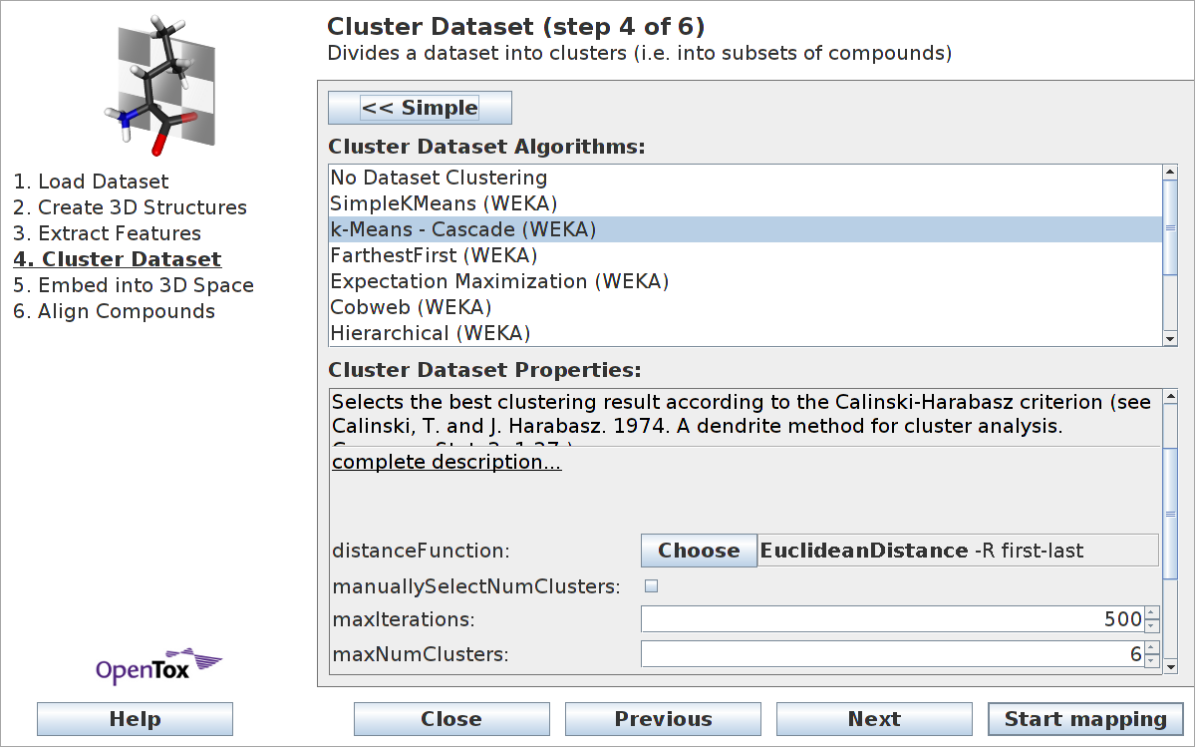
**Wizard Step 4: Cluster Dataset - advanced view**. Within the advanced view, users can choose (and configure) a cluster algorithm from the statistics library R or the data-mining library WEKA.

The *advanced *view of wizard step 4 provides a range of algorithms to choose from (see Figure 6). Cluster algorithms from the statistics library R [19], and the data-mining library WEKA [20] can be employed within CheS-Mapper. The cluster methods by R rely on a local installation of the R system on the user's computer, while the WEKA routines are built into the CheS-Mapper. Guidance on which algorithm to select can be found on the project homepage http://ches-mapper.org. The following cluster algorithms are available:

• ***k*-Means **is a basic clustering technique assigning compounds to *k *randomly initialized centroids using some distance function. Using the compounds assigned to each centroid, the centroid is re-computed, being the center of all compounds belonging to this cluster and the compounds are re-assigned to the nearest centroid. This is repeated iteratively, until the algorithm converges. This method is available in two different implementations (R and WEKA). The major difference between both implementations is that the one by R includes the concept of random restarts. In both versions, the number of clusters (*k*) has to be given by the user. This is not ideal, as the user would have to find the right number of clusters manually.

• **Cascade *k*-Means **uses a range of values for *k*, performs random restarts of the *k*-Means algorithm, and automatically selects the best value. To this end, the quality of each cluster result is evaluated with the Calinski-Harabasz criterion [21]. This method is available as R implementation [22] and as built-in implementation that employs WEKA's *k*-Means method. The latter method is the default clustering algorithm in CheS-Mapper.

• **Hierarchical clustering **is a well established cluster approach that starts by considering each compound as a single cluster and subsequently merges two clusters at a time. A distance matrix with all pair wise distances between clusters is computed, to identify the pair which is closest in distance space, that will be merged. Various different set distance schemes to compute the distance between clusters are available. In the CheS-Mapper application, hierarchical clustering is provided in two implementations, R and WEKA. Again, this method has the drawback that the number of clusters has to set to a fixed number by the user. Therefore, a dynamic version that detects the number of clusters, implemented in R, is available [23].

• **Expectation Maximization **models the data as mixture of Gaussians, i.e. each cluster is represented by one Gaussian distribution. This is a more general approach of the *k*-Means clustering that can model clusters of different spatial expansions (the Gaussians can have different standard deviations) in contrary to *k*-Means (where each compound is assigned to the closest centroid). The WEKA implementation of the EM Clustering has a built-in functionality to auto-detect the number of clusters by using cross-validation: Starting at 1, it iteratively increases the number of clusters, using the log-likelihood of the test-fold compounds as quality measure. If the log-likelihood decreases, the previous number of clusters is used.

• **Cobweb **is a hierarchical conceptual clustering algorithm, implemented in WEKA. It splits, merges, or inserts nodes in a hierarchical tree according to the category utility of a node [24].

• **Farthest First **is somewhat similar to *k*-Means, with the difference that the centroids are chosen as follows: It starts with a random data point, and chooses the point farthest from it. Subsequently, the next point that is farthest away from the already chosen points is selected until *k *points are obtained [25].

#### Embed into 3D Space

Wizard step 5 handles the 3D embedding of compounds. The embedding algorithm uses the feature values selected in step 3 as input. Accordingly, each compound is assigned a position in 3D space, such that the spatial proximity of two compounds reflects their similarity based on the features.

**Figure 7:**
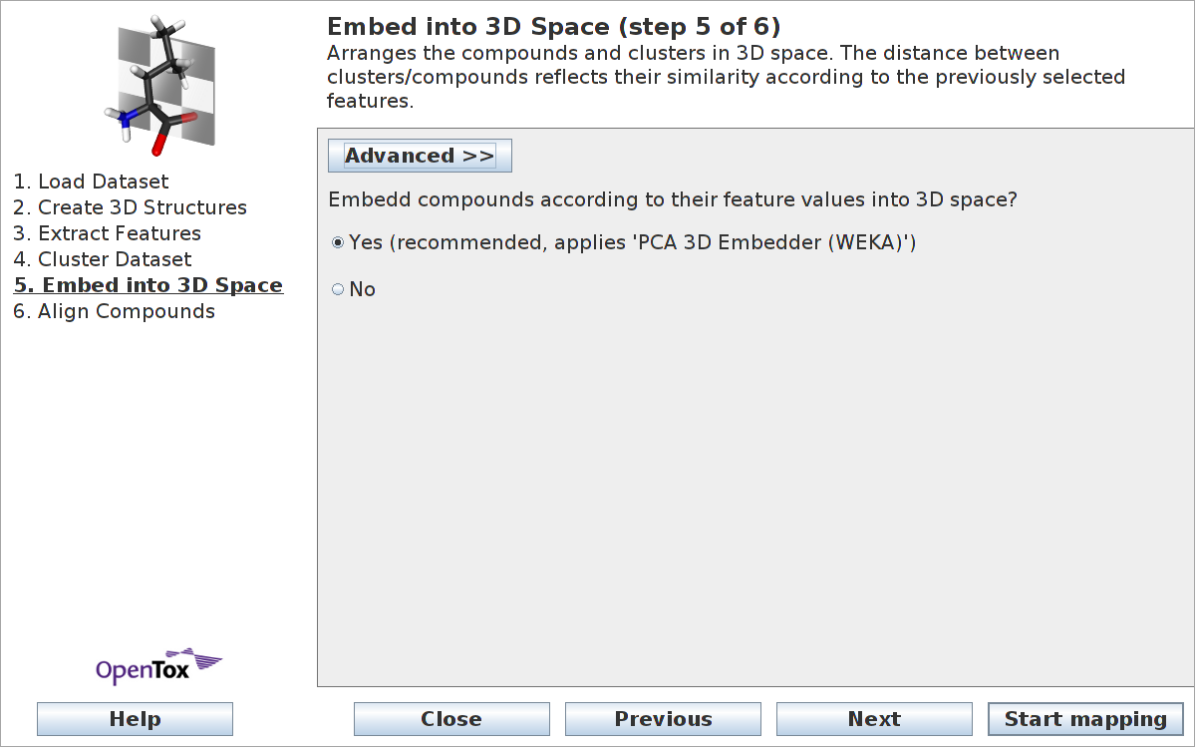
**Wizard Step 5: Embed into 3D Space - simple view**. With the simple view, users can decide whether to embed a dataset according to its features values, or not.

**Figure 8:**
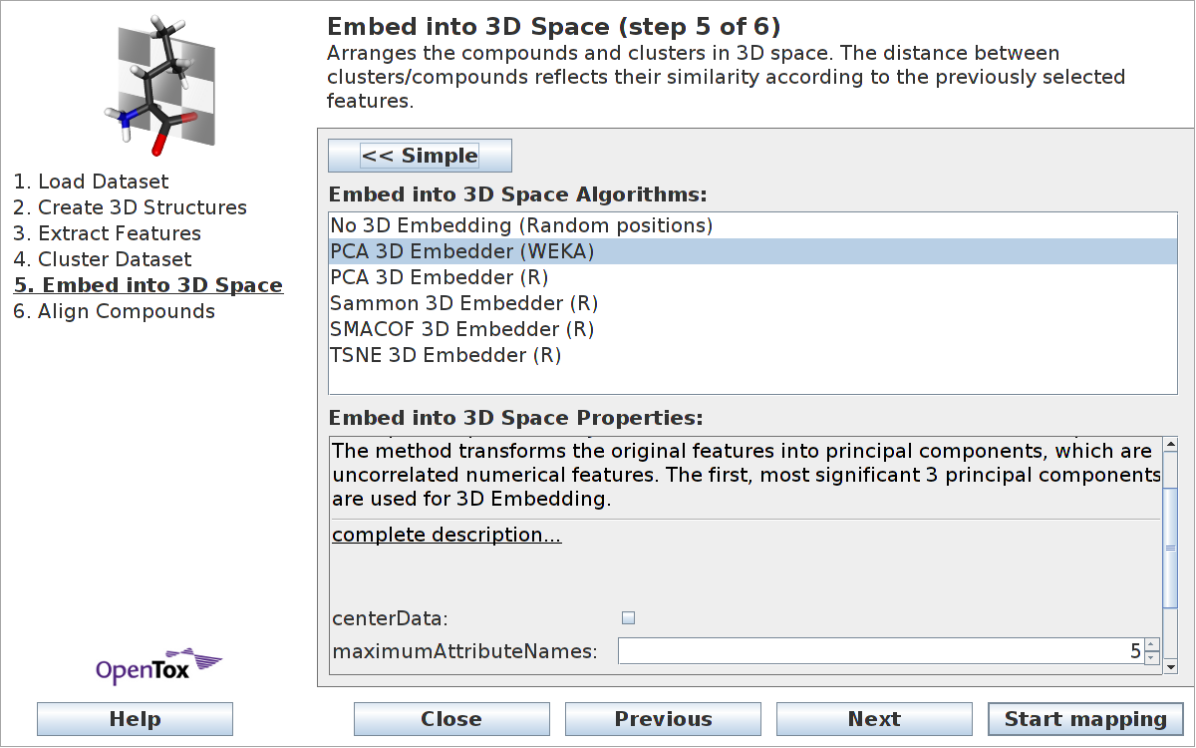
**Wizard Step 5: Embed into 3D Space - advanced view**. Within the advanced view, users can choose and configure and embedding algorithm.

Figure 7 shows the *simple *view for this wizard step: the user has to decide if the compounds should be embedded. In this case a principal component analysis is applied to determine 3D coordinates. When deciding not to embed the compounds, the compounds will be arranged at random positions in 3D space. This is the only feasible method if there are no compound features available. Guidance on which algorithm to select can be found at http://ches-mapper.org. The following embedding methods are available in the *advanced *wizard view (see Figure 8):

• **Principal component analysis (PCA) **is a method that reduces the feature space. It transforms the original features into principal components, which are uncorrelated numerical features. The first, most significant 3 principal components are used for 3D Embedding. Within CheS-Mapper, two different implementations are provided: WEKA and R. The PCA method is computationally not so expensive when compared to other embedding techniques and possesses therefore faster runtime than the methods below (see additional file 1 for runtime experiments).

• **Sammon's Non-Linear Mapping **is an iterative multidimensional scaling method [26]. The algorithms maps the high-dimensional input space to a 3D dimensional space, while trying to preserve the inherent structure of the data. The algorithm is only available as R implementation, that converges in about 50 iterations.

• **SMACOF **employs multidimensional scaling using majorization using R [27]. The algorithm is an optimization method that iteratively reduces the stress for each compound. Computing the stress values with majorization ensures a linear convergence rate. For this method, CheS-Mapper converts the feature values to a distance matrix using Euclidean distance. To reduce the runtime of this method, the user can set a maximum-number-of-iterations parameter.

• **t-distributed stochastic neighbor embedding (t-SNE) **is only available within R [28]. It is a variation of stochastic neighbor embedding that uses conditional probabilities that represent similarities. In particular, t-SNE uses a cost function that is easier to optimize (a Student-t distribution is used instead of a Gaussian to compute the similarity between two points). The parameter perplexity defines the effective number of neighbors that are used to compute the conditional probability distribution. Again, the maximum number of iterations can be reduced by the user in order to decrease the runtime.

#### Align Compounds

**Figure 9:**
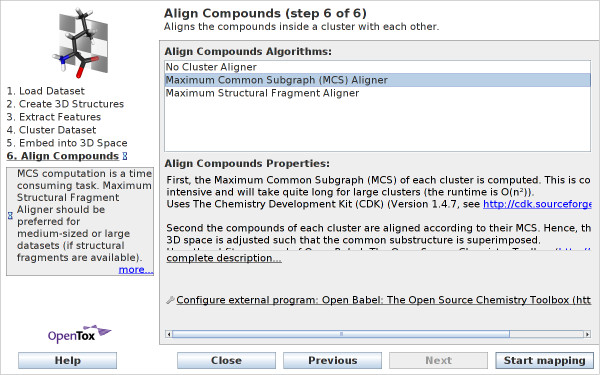
**Wizard Step 6: Align Compounds**. Users can enable 3D alignment of compounds, which is appropriate when the dataset contains structurally similar compounds.

The final wizard step allows to configure the alignment of the compounds inside a cluster according to a common substructure (see Figure 9). In other words, for each cluster there has to be a structural fragment that matches all compounds of this cluster. Two alignment methods exist that use different structural fragments:

• **Maximum common subgraph (MCS) aligner **computes the maximum common subgraph of each cluster. The CheS-Mapper program uses the CDK library for the MCS computation: CDK provides a method to find all common substructures of a molecule pair. A list of all MCS candidates is created while this method is applied subsequently to all compounds within a cluster. In cases where this procedure does not produce an MCS, no alignment is performed.

• **Maximum structural fragment (MSF) aligner **requires structural features to be selected in the *Extract Feature *wizard step. It uses the largest structural feature that matches all compounds of each cluster for alignment.

The compounds of each cluster are superimposed according to this common substructure and oriented in 3D space such that "they face the same direction". This is done with Open Babel, using the *obfit *command. Obfit accepts two compounds anda SMARTS pattern as input. It rotates the second compound such that the two matching regions in both compounds have the smallest possible root mean square deviation (RMSD). The alignment method can be used to compare the structure of structurally similar compounds that are assigned to the same cluster (see example below).

### CheS-Mapper Viewer

The viewer window of the CheS-Mapper application is based on the 3D library Jmol [29]. The mouse can be used to intuitively zoom, rotate and translate the 3D viewer. Detailed documentation on how to control the viewer can be found on the project homepage.

#### View Organization

**Figure 10:**
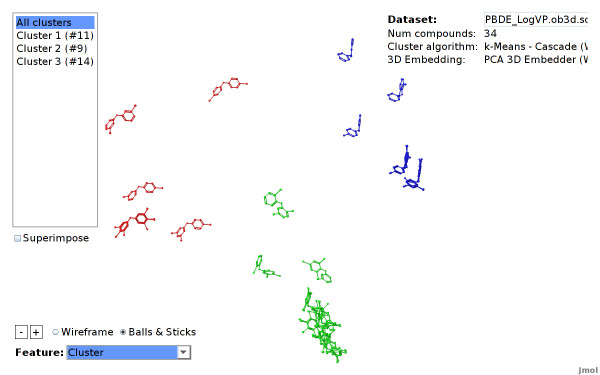
**PBDE dataset clustered**. Structural features are employed for clustering and embedding the PBDE dataset. The cluster algorithm automatically divides the dataset into 3 clusters.

When starting up, the viewer shows the complete dataset. Each compound is located at the computed 3D position and colored according to its cluster assignment (see Figure 10).

Information on what is currently displayed or selected is given on the right side of the screen. Initially, the dataset name and the dataset properties are shown, like the number of compounds, the applied cluster algorithm, and so on. Information on the selected cluster, compound or feature is added dynamically during hovering over or selecting individual compounds.

**Figure 11:**
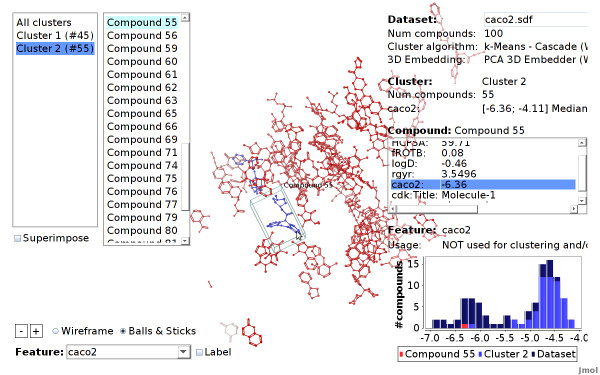
**Pirenzepine selected (Caco2 dataset)**. The compound pirenzepine is selected. It has a low endpoint value (blue color) compared to the nearby compounds (red color). The compound is an outlier considering the correlation between feature values and endpoint in this dataset.

A cluster can be selected by moving the mouse over one of the cluster compounds, or over the cluster list on the top left of the screen. The user can zoom into a cluster, by clicking on a cluster compound or on the corresponding cluster item in the list. This will hide all compounds that do not belong to this cluster. After zooming into a cluster, a single compound can be selected by simply hovering the mouse over the compound (or its corresponding item in the compound list on the top left screen). In Figure 11 the view was zoomed into a cluster, a single compound is selected. By clicking on the compound the view will further zoom to this compound.

#### Superimpose Compounds

**Figure 12:**
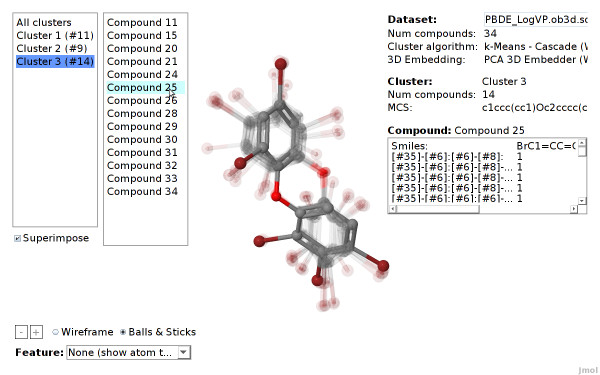
**Cluster superimposition (PBDE dataset)**. The compounds of a cluster of the PBDE are superimposed according to the maximum common subgraph of this cluster.

Superimposition moves the compounds of each cluster to the cluster center. Accordingly, they will overlap each other. This method may provide a better overview when a large clustered dataset is visualized. Furthermore, it emphasizes structural similarities of the compounds inside the cluster. This is especially useful, if the compounds of the clusters are aligned according to a common substructure (see Figure 12).

#### Highlight Clusters and Features

Initially, the compounds are highlighted according to their cluster assignment. If a single cluster is in focus (or if no clustering has been performed), the compounds are drawn in standard CPK coloring (a color convention designed by the chemists Corey, Pauling, and Koltun). The user can switch manually between the different highlight modes using the drop down menu on the bottom left of the screen.

**Figure 13:**
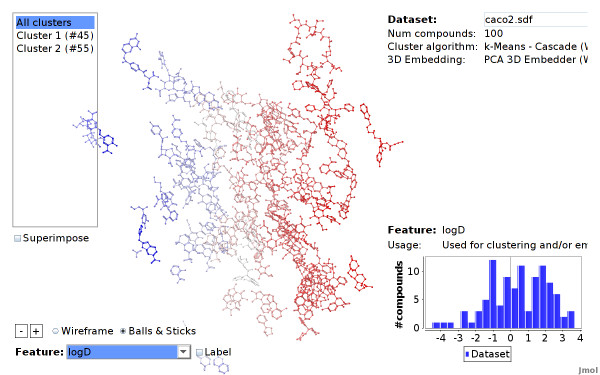
**LogD values highlighted (Caco2 dataset)**. The compounds of the Caco2 dataset, highlighted according to their LogD feature value. The embedding algorithm computes 3D positions, such that compounds with similar values are close to each other.

Additionally, features that are stored in the dataset or have been computed for clustering and embedding can be highlighted. When a feature is selected the compounds are colored according to their feature value. For numerical values, this is done withal color gradient, a continuous range of blue, white and red color. Accordingly, compounds with low feature values are colored blue, while compounds with high feature values are colored red (see Figures 13 and 11). Highlighting of nominal features assigns a distinct color to each value. Additionally, the numeric or nominal feature value of each compound can be labeled explicitly: the feature value is written next to each compound or cluster.

When using the superimpose view, the compounds of each cluster are displayed on top of each other. To retain the information provided by the highlighting, only the first compound is shown as solid, and subsequent compounds are displayed in translucent mode. The representative of each cluster can be configured in the adjacent drop down menu: the user can select *Maximum*, *Median*, or *Minimum*. Hence, the compound with the highest/median/minimum feature value is painted solid, and the remaining compounds are sorted accordingly.

When a specific feature is selected, a chart showing the histogram of the feature values is displayed in the bottom right corner. When a cluster and/or a compound is selected, their feature values are indicated in the chart (see Figure 11).

**Figure 14:**
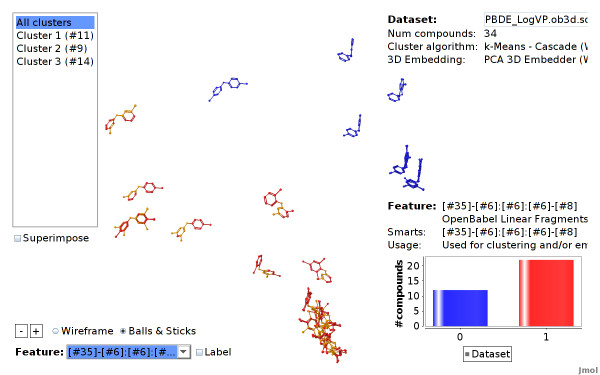
**Feature "Br-c:c:c-O" matched (PBDE dataset)**. The compounds of the PBDE dataset, highlighted according to the structural fragment "Br-c:c:c-O". The matching atoms in each compounds are drawn in orange.

As described above, a feature may represent a structural fragment encoded as a SMARTS string. When such a structural feature is selected, the SMARTS structure is matched and highlighted in the corresponding compounds (see Figure 14). Additionally, if cluster alignment according to a common fragment is enabled, this fragment can be highlighted as well.

#### Adjust the Size of the 3D Space

In order to get a more detailed and closer view on compounds or clusters, the user can use the zooming function. This will show a smaller section of the whole dataset in larger scale.

Additionally, there is the possibility to change the size of the 3D space, which affects the distance between compounds to each other (by clicking the "+" or "-" buttons on the bottom left of the screen). This can be advantages, as sometimes compounds overlap in the field of view, enlarging the 3D space overcomes that effect.

#### Export or Remove Clusters and Compounds

The user can remove compounds or whole clusters from the view, not modifying the original dataset. However, the left over compounds or clusters can then be exported as SD-file. Within the SD-file all data is stored, including the 3D structure, cluster assignments, and computed features.

### Implementation

The CheS-Mapper is implemented in Java. It is provided as a Java Web Start application, which can be directly be started from a web browser. Additionally, the program is also available as stand-alone version. CheS-Mapper is an open source project hosted at git-hub. The code architecture is interface driven, so developers can easily integrate novel algorithms for e.g. clustering, 3D structure calculation, et cetera. A range of Java libraries is integrated within the project: the 3D viewer for molecules Jmol [29], the Chemical Development Kit (CDK [11]), and the data mining workbench WEKA [20].

Extended functions are provided for cases the following software tools already pre-installed on the local computer (both freely available):

• **Open Babel **is a C++ library for chemoinformatics [15] (Open Babel is used by CheS-Mapper for 3D structure computing, SMARTS matching, structural fragment mining, and 3D alignment).

• **R **is a tool for statistical computing [19] (R is used by CheS-Mapper for clustering and embedding).

## Use Cases - Applying CheS-Mapper to Real World Datasets

### Mapping a Dataset using Integrated Features

We use the CheS-Mapper application to visualize and verify work on the correlation of Caco-2 permeation with simple molecular properties [30]. The authors provide 100 structural diverse compounds, with five numeric features that are stored in the dataset. One of the features is the actual endpoint Caco-2 permeability (*logP_app_*). The remaining four molecular descriptors are: experimental distribution coefficient (*logD*), high charged polar surface area (*HCPSA*), radius of gyration (*rgyr*), and fraction of rotatable bonds (*fROTB*). In their work, the authors describe, that these four features are valuable descriptors for building a QSAR model to predict Caco-2 permeation.

We employ the CheS-Mapper wizard to embed the compounds in 3D space according to those four properties using principal components analysis. Note that the actual endpoint (*caco2*) was not used. A copy of the dataset and a detailed tutorial can be found at http://ches-mapper.org.

**Figure 15:**
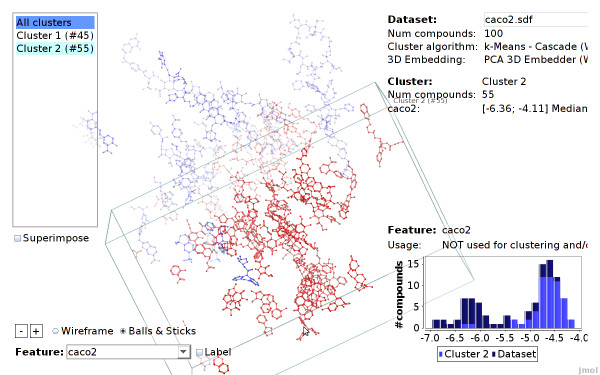
**Endpoint values highlighted (Caco2 dataset)**. The compounds of the Caco2 dataset, highlighted according to their endpoint value. Compounds with similar endpoint values are close to each other, even though the endpoint was not used for embedding.

Figure 13 shows a screenshot of the CheS-Mapper viewer, with *LogD *values highlighted: the compounds are colored according to their feature value of *LogD*, compounds with low values are colored in blue, compounds with high values are colored in red. As this feature was used for embedding, compounds with similar values are close to each other. The same holds if we select the remaining three features. In contrary, the actual endpoint was not used as input for the embedding algorithm. Still, compounds that are close to each other tend to have a similar *caco2 *value (see Figure 15). This supports the authors findings, the endpoint is indeed correlated to the feature values presented in the dataset.

Additionally, we easily detected one compound with the Viewer that violates the correlation between feature values and endpoint: Figure 11 shows the compound *pirenzepine*. It has a relatively low endpoint value of -6.35, and is therefore drawn in blue. This compound attracts our attention, as it is close to compounds with high endpoint values, i.e. it is located next to many red compounds. By employing CheS-Mapper, we effortlessly gained insights on dataset and a critical compound: pirenzepine is the training compound with the highest prediction error using the QSAR model in the cited article.

### Structural Clustering using Open Babel Fingerprints

We use the CheS-Mapper program to apply structural fragment mining to a small dataset with structural similar compounds. The dataset consists of 34 polybrominated diphenyl ethers (PBDEs) with experimentally measured endpoint (*Vapor pressure*) iteester-papa. It was used to build and validate quantitative structure-property relationship (QSPR) models with physico-chemical descriptors (computed with the Dragon software). The authors determined the feature *T(O...Br) *to be the most significant descriptor with respect to the endpoint. This feature describes the sum of the topological distance between oxygen and bromine. This can be visually verified with CheS-Mapper.

We select linear fragments (up to a size of 7 atoms) as features in the wizard. We skip uninformative fragments that occur in each compound of the dataset, which yields 15 distinctive fragments, all containing bromine. Note that the actual target endpoint is not selected for clustering and embedding. Again, a copy of the dataset and a detailed tutorial can be found on the project homepage.

**Figure 16:**
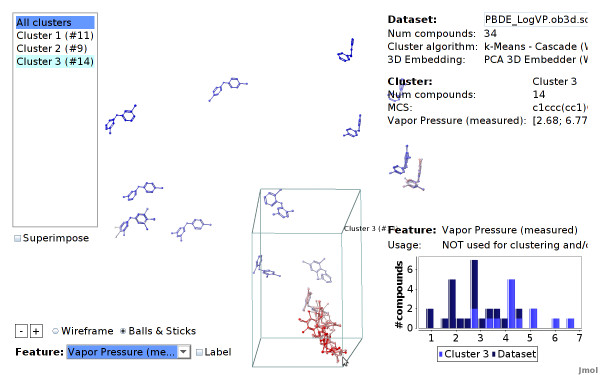
**Endpoint values highlighted (PBDE dataset)**. The compounds of the PBDE dataset, highlighted according to their endpoint value. The selected cluster contains the compounds with the highest endpoint values. Hence, the endpoint values depend on the compound structure.

The viewer shows that there are three clusters of about equal size (see Figure 10). We highlight the endpoint in Figure 16 to show that the endpoint can in fact be modeled with these 15 structural features: features that are located next to each other have a similar endpoint values. Furthermore, one of the clusters contains all compounds with high endpoint values.

We now determine the feature properties of the three clusters. We select the structural feature *[#35]-[#6]:[#6]:[#6]-[#8] *that corresponds to a linear sequence of 5 atoms: bromine, three aromatic carbons, and oxygen (the SMARTS is equal to *ur-c:c:c-O*). Figure 14 shows that the CheS-Mapper viewer colors the compounds according to occurrence, as well as it highlights where the smarts matches in each compound. Selecting feature *Br-c:c-O *shows that the compounds in cluster number three (the one with the highest endpoint values) exclusively match both fragments. This supports the findings [31], that the endpoint value depends indeed on the on the distance between the bromine atom and the ether group.

CheS-Mapper can be a valuable tool for structurally very similar compounds, when employing MCS alignment in together with the superimpose mechanism. Figure 12 shows the compounds of cluster number three superimposed onto each other. The compounds are aligned according to their common substructure, enabling the user to identify structural similarities or dissimilarities.

## Conclusions

We present the CheS-Mapper application, a tool to visualize and explore chemical datasets. In a preprocessing step, the dataset is mapped into a virtual three-dimensional space. A key part of the preprocessing is the choice of features, which is done by the user. Features can either be provided in the dataset, or the application can calculate physico-chemical descriptors as well as structural fragments. The selected features are then used for clustering and 3D embedding. Hence, compounds that have similar feature values are likely to be clustered together, and are close to each other in 3D space. Subsequently, a 3D viewer shows the embedded dataset, and enables the user to explore the dataset and its properties. Numerical features, as well as structural features can be highlight within the viewer.

This makes CheS-Mapper a visualization tool that could be used to explore structure-activity relationship (SAR) information in datasets, as well as to present chemical compounds and compound features to others. It is freely available and an open-source project.

## Availability and requirements

• **Project name: **CheS-Mapper

• **Project home page: **http://ches-mapper.org

• **Operating system(s): **Cross-platform

• **Programming language: **Java with Java Web Start support (can be started from a web browser)

• **Other requirements (optional): **For extended functions Open Babel [15] and R [19] (both free).

• **License: **GNU GPL v3

• **Any restrictions to use by non-academics: **None. For proper use, guidance and maintenance, contact ches-mapper@informatik.uni-freiburg.de

## Competing interests

The authors declare that they have no competing interests.

## Authors' contributions

MG implemented and designed the CheS-Mapper software. AK and SK provided the idea for CheS-Mapper as well as valuable guidance throughout the implementation. All authors read and approved the final manuscript.

## Supplementary Material

Additional file 1 **Supplementary Material for the article**. CheS-Mapper - Chemical Space Mapping and Visualization in 3D. Click here for file
