# Supplementary material for: CheS-Mapper - Chemical Space Mapping and Visualization in 3D
Source: J Cheminform. 2012 Mar 17;4:7. doi: 10.1186/1758-2946-4-7 (PMC3331825; doi:10.1186/1758-2946-4-7)
Supplement: Additional file 1 — Supplementary Material for the article. CheS-Mapper - Chemical Space Mapping and Visualization in 3D. [file 1758-2946-4-7-S1.PDF]

Supplementary Material for the article:
CheS-Mapper – Chemical Space Mapping and Visualization in 3D

Submitted to the Journal of Cheminformatics

Martin Gütlein[1], Andreas Karwath[1], Stefan Kramer[2]
[1] Department of Computer Science, Albert-Ludwigs-Universität Freiburg, Germany
[2] Institute for Computer Science, Johannes Gutenberg-Universität Mainz, Germany

The following supplementary material is available on our project homepage http://ches-mapper.org as well.

Supported Dataset Formats and Size

Size

We successfully tested CheS-Mapper with datasets with over 6000 compounds, however pre-processing takes quite a while on these datasets (see runtime below). We recommend CheS-Mapper for datasets of up to 1500 compounds.

Formats (with corresponding file ending)

CTX (ctx), PubChem Substances ASN (asn), Mol2 (Sybyl) (mol2), MDL Molfile V2000 (mol), Gaussian94, CrystClust (crystclust), PubChem Compound XML (xml), IUPAC-NIST Chemical Identifier (XML) (inchi), Gaussian92, PolyMorph Predictor (Cerius) (pmp), Crystallographic Interchange Format (cif), PubChem Substance XML (xml), Gaussian 2003, Gaussian95, Q-Chem (qc), Jaguar (j), Aces2, Ghemical Quantum/Molecular Mechanics Model (gpr), MoSS Output Format (mossoutput), MOPAC 2002 (mop), MDL Molfile (mol), CACheMolStruct (cache), MDL Reaction format (rxn), Ghemical Simplified Protein Model, Gaussian90, MOPAC7 (mop), MDL Structure-data file (sdf), MOPAC 97 (mop), Protein Brookhave Database (PDB) (pdb), ZMatrix, VASP, ADF, PubChem Compound ASN (asn), IUPAC-NIST Chemical Identifier (Plain Text), MDL Mol/SDF V3000, Spartan Quantum Mechanics Program, PubChem Substances XML (xml), NWChem (nw), HyperChem HIN (hin), PubChem Compounds XML (xml), GAMESS log file (gam), Dalton, ABINIT, ShelXL (ins), MDL RXN V3000 (rxn), Chemical Markup Language (cml), Symyx Rgroup query files (mol), Gaussian98, CDK OWL (N3) (n3), MOPAC 93 (mop)

Algorithm Runtimes

The experiments have been performed on an Intel(R) Core(TM)2 Duo CPU P9500 with 2.53GHz with 4G main memory. (The dual core does not affect the runtime though, as CheS-Mapper is currently not designed to make use of multiple cores.)

Datasets

Dataset	Compounds	Description	Source
PBDE	34	Polybrominated diphenyl ethers (endpoint 'Vapor pressure')	http://onlinelibrary.wiley.com/doi/10.1002/qsar.200860183/abstract
caco2	100	Diverse compounds tested for Caco-2 permeability	http://pubs.acs.org/doi/suppl/10.1021/ci049884m
cox2	467	Cyclooxygenase-2 (COX-2) inhibitors	http://pele.farmbio.uu.se/qsar-ml/qsarml-datasets.html
CPDBAS	1508	Carcinogenic Potency Database - All Species	http://www.epa.gov/ncct/dsstox/sdf_cpdbas.html

Create 3D Structures

Dataset	Compounds	CDK	OpenBabel
PBDE	34	5s	2m, 21s
caco2	100	4m, 5s	20m, 12s
cox2	467	1m, 22s	1h, 40m
CPDBAS	1508	28m, 32s	*not tested*

Building 3D structures of compounds is a time consuming task (the energy of each compound is minimized using an iterative optimization method). You have to do this only once (results are cached by CheS-Mapper).

Extract Features

Dataset	Compounds	CDK constitutional	CDK all*	All OpenBabel fingerprints	52 Smarts** OpenBabel	52 Smarts** CDK
PBDE	34	4s	7s	3s	1s	3s
caco2	100	9s	1m, 17s	2s	<1s	4s
cox2	467	38s	4m, 33s	5s	1s	24s
CPDBAS	1508	2m, 4s	19m, 18s	14s	5s	39s

CDK all*

All CDK descriptors, except Ionization Potential (takes about 5 seconds per compound)

52 Smarts**

52 structural alerts extracted from ToxTree plugin: Benigni / Bossa rulebase (for mutagenicity and carcinogenicity)

Features for clustering and embedding

The clustering and embedding algorithms have been used twice on each dataset, with different feature sets each:

- Using all CDK features (without Ionization Potential, this produces 274 regression numeric features).
- Using OpenBabel Linear Fragments (min-frequency set to 5% of the dataset size), this produces a couple hundred binary features.

Cluster Dataset

Dataset	Size	Features	Simple k-Means (WEKA)	k-Means - Cascade (WEKA) *	k-Means - Cascade (WEKA) **	Farthest First (WEKA)	EM (WEKA) ***	EM (WEKA) ****	Cobweb (WEKA)	Hierarch (WEKA)	k-Mean s (R)	k-Means - Cascade (R)	Hierarch (R)	Hierarch - Dynamic Tree Cut (R)
PBDE	34	274 CDK	<1s	<1s	<1s	<1s	2s	<1s	<1s	<1s	<1s	<1s	<1s	<1s
caco2	100	274 CDK	<1s	3s	<1s	<1s	5s	<1s	<1s	<1s	<1s	1s	<1s	<1s
cox2	467	274 CDK	<1s	35s	1s	<1s	5m, 25s	3s	<1s	2s	1s	7s	<1s	1s
CPDBAS	1508	274 CDK	5s	4m, 36s	22s	1s	*not tested*	29s	5s	42s	5s	59s	19s	17s

Dataset	Size	Features	Simple k-Means (WEKA)	k-Means - Cascade (WEKA) *	k-Means - Cascade (WEKA) **	Farthest First (WEKA)	EM (WEKA) ***	EM (WEKA) ****	Cobweb (WEKA)	Hierarch (WEKA)	k-Mean s (R)	k-Means - Cascade (R)	Hierarch (R)	Hierarch - Dynamic Tree Cut (R)
PBDE	34	15 LinFrag (f=1)	<1s	<1s	<1s	<1s	<1s	<1s	<1s	<1s	<1s	4s	<1s	<1s
caco2	100	450 LinFrag (f=5)	<1s	21s	2s	<1s	2m, 46s	2s	1s	<1s	<1s	3s	<1s	<1s
cox2	467	416 LinFrag (f=23)	<1s	58s	5s	<1s	25m, 23s	8s	11s	1s	1s	14s	2s	1s
CPDBAS	150 8	201 LinFrag (f=75)	4s	2m, 55s	19s	1s	*not tested*	30s	1m, 4s	16s	2s	36s	21s	15s

k-Means - Cascade (WEKA)*

This is the default clusterer (using the simple view of the cluster wizard step). It repeats Simple k-Means with k values 2 to 10, and makes 10 random restarts each.

k-Means - Cascade (WEKA)**

The same clusterer using k values 3 to 5 with 3 restarts each. This is faster than the previous setting.

Expectation Maximization (WEKA)***

This is the EM cluster algorithm with default settings (numClusters = -1). This performs an internal cross-validation to determine the best number of clusters, which may be time consuming.

Expectation Maximization (WEKA)****

The EM cluster algorithm with a fixed number of clusters (k), much faster than the previous setting.

Embed into 3D Space

Dataset	Compounds	Features	PCA 3D Embedder (R)	r^2	PCA 3D Embedder (WEKA)	r^2	Sammon 3D Embedder (R)	r^2	SMACOF 3D Embedder (R)*	r^2	SMACOF 3D Embedder (R)**	r^2
PBDE	34	274 CDK	<1s	0.99	<1s	0.94	<1s	0.98	46s	0.99	13s	0.88
caco2	100	274 CDK	<1s	0.48	<1s	-0.01	<1s	0.58	37m, 21s	-0.00	7m, 37s	0.07
cox2	467	274 CDK	<1s	-0.02	<1s	-0.90	2s	-2.55	*not tested*	-	*not tested*	-
CPDBAS	1508	274 CDK	2s	0.61	2s	-0.35	29s	0.60	*not tested*	-	*not tested*	-

Dataset	Compounds	Features	PCA 3D Embedder (WEKA)	r^2	PCA 3D Embedder (R)	r^2	Sammon 3D Embedder (R)	r^2	SMACOF 3D Embedder (R)*	r^2	SMACOF 3D Embedder (R)**	r^2
PBDE	34	15 LinFrag (f=1)	<1s	0.79	1s	0.82	4s	0.88	2s	0.83	1s	0.82
caco2	100	450 LinFrag (f=5)	3s	-6.64	<1s	-4.14	<1s	-5.09	1m, 13s	-5.01	16s	-5.34
cox2	467	416 LinFrag (f=23)	10s	-0.37	1s	0.24	8s	-1.08	*not tested*	-	6m, 7s	-0.10
CPDBAS	1508	201 LinFrag (f=75)	8s	-2.46	1s	-0.92	1m, 6s	-6.17	*not tested*	-	*not tested*	-

SMACOF 3D Embedder (R)*

The SMACOF embedder used with default settings (number of iterations = 150)

SMACOF 3D Embedder (R)**

The SMACOF embedder used with number of iterations = 30, to ensure a faster runtime

Align Compounds

Dataset	Compounds	Number of Clusters*	Maximum Common Subgraph (MCS) Aligner	Maximum Structural Fragment Aligner
PBDE	34	3	1s	<1s
caco2	100	5	8s	1s
cox2	467	6	4s	2s
CPDBAS	1508	6	2s	2s

Number of Clusters*

We clustered the datasets using the Dynamic Tree Cut clusterer based on structural features (OB Linear Fragments as above)
